# The Starch Is (Not) Just Another Brick in the Wall: The Primary Metabolism of Sugars During Banana Ripening

**DOI:** 10.3389/fpls.2019.00391

**Published:** 2019-04-02

**Authors:** Beatriz Rosana Cordenunsi-Lysenko, João Roberto Oliveira Nascimento, Victor Costa Castro-Alves, Eduardo Purgatto, João Paulo Fabi, Fernanda Helena Gonçalves Peroni-Okyta

**Affiliations:** ^1^ Department of Food Science and Experimental Nutrition, School of Pharmaceutical Sciences, University of São Paulo, São Paulo, Brazil; ^2^ Food Research Center (FoRC), Research, Innovation and Dissemination Centers, São Paulo Research Foundation (CEPID-FAPESP), São Paulo, Brazil; ^3^ Food and Nutrition Research Center (NAPAN), University of São Paulo, São Paulo, Brazil

**Keywords:** starch-degrading enzymes, starch degradation, starch ultrastructure, banana, ethylene, ripening, sucrose

## Abstract

The monocot banana fruit is one of the most important crops worldwide. As a typical climacteric fruit, the harvest of commercial bananas usually occurs when the fruit is physiologically mature but unripe. The universal treatment of green bananas with ethylene or ethylene-releasing compounds in order to accelerate and standardize the ripening of a bunch of bananas mimics natural maturation after increasing the exogenous production of ethylene. The trigger of autocatalytic ethylene production regulated by a dual positive feedback loop circuit derived from a NAC gene and three MADS genes results in metabolic processes that induce changes in the primary metabolism of bananas. These changes include pulp softening and sweetening which are sensorial attributes that determine banana postharvest quality. During fruit development, bananas accumulate large amounts of starch (between 15 and 35% w/w of their fresh weight, depending on the cultivar). Pulp softening and sweetening during banana ripening are attributed not only to changes in the activities of cell wall hydrolases but also to starch-to-sugar metabolism. Therefore, starch granule erosion and disassembling are key events that lead bananas to reach their optimal postharvest quality. The knowledge of the mechanisms that regulate sugar primary metabolism during banana ripening is fundamental to reduce postharvest losses and improve final product quality, though. Recent studies have shown that ethylene-mediated regulation of starch-degrading enzymes at transcriptional and translational levels is crucial for sugar metabolism in banana ripening. Furthermore, the crosstalk between ethylene and other hormones including indole-3-acetic acid and abscisic acid also influences primary sugar metabolism. In this review, we will describe the state-of-the-art sugar primary metabolism in bananas and discuss the recent findings that shed light on the understanding of the molecular mechanisms involved in the regulation of this metabolism during fruit ripening.

## Banana Starch Structure

During development, banana (*Musa acuminata*) fruit accumulates a large reserve of carbon in the form of starch. At the 3/4 diameter stage, which is considered to be the optimum point for commercial harvesting of bananas, the fruits have 12–35% starch, while the starch content at late ripening usually ranges from 15% to less than 1% (Soares et al., 2011). The mobilization of the starch reserve is followed by a concomitant increase in soluble sugars that may reach up to 20% of the fresh weight of the pulp in the ripe fruit, with sucrose accounting for approximately 80% of the soluble sugars in ripe bananas, whereas glucose and fructose make up almost all the remaining 20% of the soluble sugars in equal proportions (Henderson et al., 1959; Marriott et al., 1981; Hill and ap Rees, 1993; Cordenunsi and Lajolo, 1995; Mota et al., 1997; Shiga et al., 2011). However, maltose and other oligosaccharides, such as a trisaccharide correlate to invertase (INV) activity (Henderson et al., 1959), and fructooligosaccharides (FOS) (Der Agopian et al., 2008) are also detected during banana ripening. In this regard, the presence of 1-kestose, the first member of the FOS series, occurs when the levels of sucrose in the pulp reach 200 mg/g of dry matter, and an INV appears to synthesize the FOS by transfructosylation (Der Agopian et al., 2008).

The amount of starch in the fully developed fruit may vary significantly among banana varieties belonging to different species. Compared to Cavendish bananas (*M. acuminata*), the cooking varieties classified as plantains (*M. × paradisiaca*) accumulate more starch (up to 35%) and have larger amounts of undegraded starch when ripe, which means they are not as sweet as Cavendish bananas. As an example, the fully ripe plantain Brazilian cultivars (cv.), Terra (genotype group AAB) and Figo (genotype group ABB), have 8–16% and 6–9% residual amounts of starch and sucrose, respectively (Soares et al., 2011), which are equivalent to those values observed for plantains grown in Ghana and some dessert bananas purchased in the UK (Marriott et al., 1981). In contrast, the dessert bananas cv. Pacovan and Mysore (genotype group AAB) have approximately 1% of residual starch (Soares et al., 2011). Therefore, there is an indication that the pattern of starch accumulation and degradation is highly correlated with the banana specie.

Starch consists of linear amylose and highly branched amylopectin in the proportion 20:80. Amylose is formed by linear α-D-(1,4)-glucose units, whereas amylopectin consists of several short chains of α-D-(1,4)-glucose units interconnected by α-D-(1-6)-glucose units making up to 6% of the bonds in the molecule (Buléon et al., 1998; Hoover, 2001; BeMiller, 2019). These two macromolecules are arranged in the form of granules with a well-organized internal structure, alternating between semi-crystalline and amorphous layers, which are also known as growth rings.

The morphology of the starch granules differs according to the botanical origin, the cultivar of fruits, and the ripening stage (Peroni-Okita et al., 2010, 2013, 2015; Soares et al., 2011). Starch granules from unripe bananas were found to be oval and rounded for cv. Nanicão and small and leaf-like shaped for cv. Pacovan and Mysore. In plantains, the starch granules from unripe fruits were mostly round and elongated for cv. Terra and Figo, but some oval and rounded starch granules were present in the unripe fruit. In contrast, the partially degraded starch granules from both overripe bananas and plantains were narrow and elongated (Soares et al., 2011). Thus, as the small and round granules were degraded in bananas during ripening and almost disappeared in ripe plantains, it appears that granules with this shape and size have a greater susceptibility to enzymatic degradation (Peroni-Okita et al., 2010; Soares et al., 2011). Supporting this hypothesis, Gao et al. (2016) also reported rounded granules for unripe Cavendish varieties and smaller and ellipsoid granules in ripe plantains, which changed during ripening to an irregular shape in the first group of bananas and remained unchanged as ellipsoidal granules in the last group. As expected, starch granules appear decreasing in size during ripening. The size distribution analysis revealed that the granules from unripe bananas of cv. Nanicão averaged 28.9 μm, with 90% of the population being smaller than 49.6 μm and 10% being less than 10.3 μm. The granules from ripe fruits were smaller (25.4 μm), and the size distribution showed that 90% of the population was 45.4 μm and 10% was 7.6 μm (Peroni-Okita et al., 2010).

The investigation of starch granule surface during banana ripening, using microscopic and physical techniques, revealed that the inner part of the granule consists of a material with different viscoelastic properties (Peroni-Okita et al., 2010, 2013, 2015; Soares et al., 2011). In ripe bananas, the inner part of the granule is composed predominantly from large blocklets (80–200 nm), which consist of amylopectin lamellae with spherical structures that absorb less water than other parts. In contrast, green bananas have smaller blocklets (15–50 nm) in the inner part of the granule (Peroni-Okita et al., 2015).

The organization of the starch granule depends on the packing of amylopectin double helices; those from cereals, mango, and tapioca usually have the A-type pattern, which is associated with a monoclinic lattice with densely packed crystallites. In contrast, the granules from tubers and high amylose content starches have the B-type pattern composed of hexagonal crystalline unit cells that contain much more water (Gallant et al., 1997; Buléon et al., 1998; Thys et al., 2008). Banana starch granules show a typical C-type profile, resulting from the coexistence of A- and B-type allomorphs in the same granule (Millan-Testa et al., 2005; Peroni-Okita et al., 2010; Soares et al., 2011).

During banana ripening, the crystallinity index of starch granules decreases, although the total amylose content remained almost constant. Since the short chains of amylopectin are reduced, and the degree of crystallinity is dependent on the proportion of amylopectin, the amount of short A-type chains plays a role in the polymorphic forms of starch crystals. In fact, the amylopectin from cv. Nanicão has a large amount of short A- and B1-type chains and a reduced amount of long B-type chains. The ratios of the fraction fa/fb1 + fb2 + fb3 decreased during ripening. These fractions of amylopectin correspond to A-chains (fa, external short chains, DP 6–12), B1 (fb1, DP 13–24), B2 (fb2, DP 25–36), and long B3 chains (fb3, DP > 37), and the ratio indicates the length and the degree of ramification of amylopectin chains (Hanashiro et al., 1996). Large proportions of short chains suggest a more crystalline starch granule. This clearly demonstrates that the degree of crystallinity is dependent on the branching patterns of amylopectin and may play an important role in determining the type of unit packing, the wide-angle X-ray diffraction (WAXD) pattern, and the susceptibility to enzymatic hydrolysis (Jane et al., 1997; Sanderson et al., 2006).

The amylose content in starch granule also plays an important role in accessibility to degrading enzymes, as the double helices formed by amylose acquire resistance to amylase hydrolysis. In general, the amylose content is greater in plantain than in Cavendish or dessert varieties and significantly decreases during ripening (Zhang et al., 2005; Peroni-Okita et al., 2010; Shiga et al., 2011; Chavez-Salazar et al., 2017). In this regard, the decrease in amylose content in starch granules from bananas cv. Figo during ripening appears to occur through the exo-corrosion of the amylose-rich layers of a granule population that is more susceptible to degradation. According to Soares et al. (2011), the small and round granules almost disappear at the same time that the A/B-type allomorph ratio reduces (2.03–1.27) during ripening, suggesting that the more susceptible round starch granules are predominantly A-type. Therefore, the application of atomic force microscopy (AFM), scanning electron microcopy (SEM), and WAXD to the analysis of starch granules from cv. Terra and cv. Nanicão demonstrated that the subtle changes observed at the surface were related to the lamellar organization of starch, suggesting that A-type crystallites located at the periphery of starch granules are preferentially degraded during ripening (Soares et al., 2011; Peroni-Okita et al., 2015). The granules of banana starch are highly resistant to enzymatic hydrolysis, and they appear to undergo the natural process of degradation by enzymatic corrosion of the surface, in a layer-by-layer process. Furthermore, the abovementioned studies show no evidence of porous structures at the granule surface, which would facilitate the access of hydrolases during ripening.

**Figure 1:**
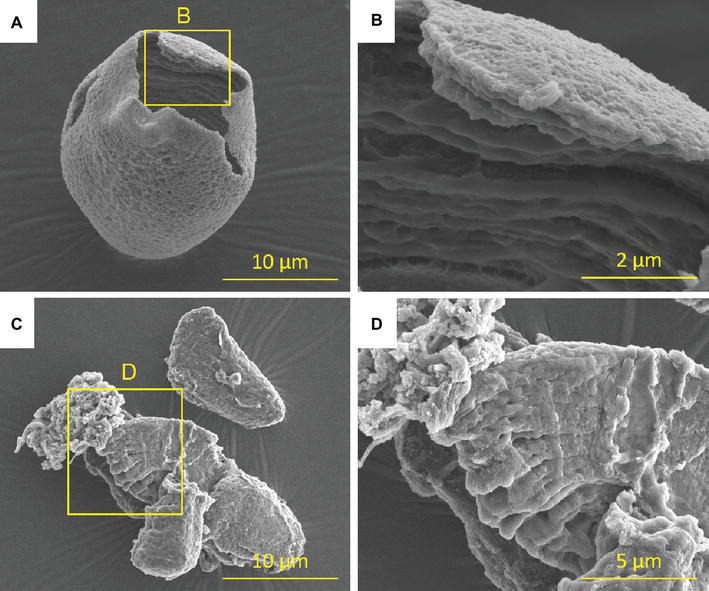
Scanning electron microscopy (SEM) images of starch from unripe bananas. Starch granules isolated from unripe **(A)** Terra (plantain), **(B)** magnification of image, **(C)** Thap Maeo (banana), and **(D)** magnification of image, treated with porcine α-amylase (24 h; 37°C; 3 U/mg of starch). Images produced by SEM were acquired by the authors following the protocol described by Peroni-Okita et al. (2015).

Data from AFM analysis also support the idea that the first layer covering the granule surface is composed of a hard or well-organized material. The removal of this first layer exposes new layers with alternate hard and soft regions repeated at regular intervals until a hard and well-organized semi-crystalline growth ring is attained. Results obtained by Peroni-Okita et al. (2010) and Soares et al. (2011) have suggested that this first layer is more resistant to the enzymes that degrade the starch granule in starches isolated from plantains than in Cavendish. Figure 1 shows an interesting result obtained when granules isolated from cv. Terra (plantain) and Thap Maeo (Cavendish) were treated with amylase porcine for several hours. Some granules were very corroded in the interior, with the remaining shell being almost integrated in the case of cv. Terra (Figures 1A,B), which was not seen in cv. Thap Maeo (Figures 1C,D). According to Peroni-Okita et al. (2015), no migration of enzymes from the surface or signs of degradation was observed in the inner part of starch granules from unripe bananas, suggesting that the layers are more resistant to enzymatic corrosion. Since the core of the granules was weakly stained with iodine, the amount and distribution of amylose chains in the center of the particle were likely to be an important contributor to the resistance of banana starch to hydrolysis, alongside several other factors (Gallant et al., 1992, 1997; Faisant et al., 1995; Jiang et al., 2015).

## Starch-to-Sucrose Metabolism During Banana Ripening

During banana development, a large amount of starch accumulates in the amyloplasts of cells from fruit pulp (Beck and Ziegler, 1989; Nascimento et al., 2000; Mota et al., 2002). However, during banana ripening, a complex regulatory mechanism shifts metabolism from starch synthesis to starch breakdown leading to the accumulation of soluble sugars, mainly sucrose, that will have a significant impact in fruit taste and flavor. This starch-to-sucrose conversion appears to be responsible not only for pulp sweetening but also for providing energy to metabolic processes that result in the development of other quality attributes of ripe bananas, such as color change, synthesis of volatile compounds, and even pulp softening, thereby strongly affecting final fruit quality. In this sense, the disappearance of the large stock of starch in favor of the accumulation of soluble sugars strongly contributes to pulp softening (Shiga et al., 2011).

Starch-to-sucrose metabolism has been extensively studied in model systems in the context of energy sources for plant growth and development, including *Arabidopsis* leaves (transitory starch) and the endosperm of germinating cereal seeds (storage starch). Both metabolism and energy supply in photosynthetic tissues clearly differ from the equivalent processes in heterotrophic tissues.

The starch breakdown in fleshy fruits such as bananas is less understood.

**Figure 2:**
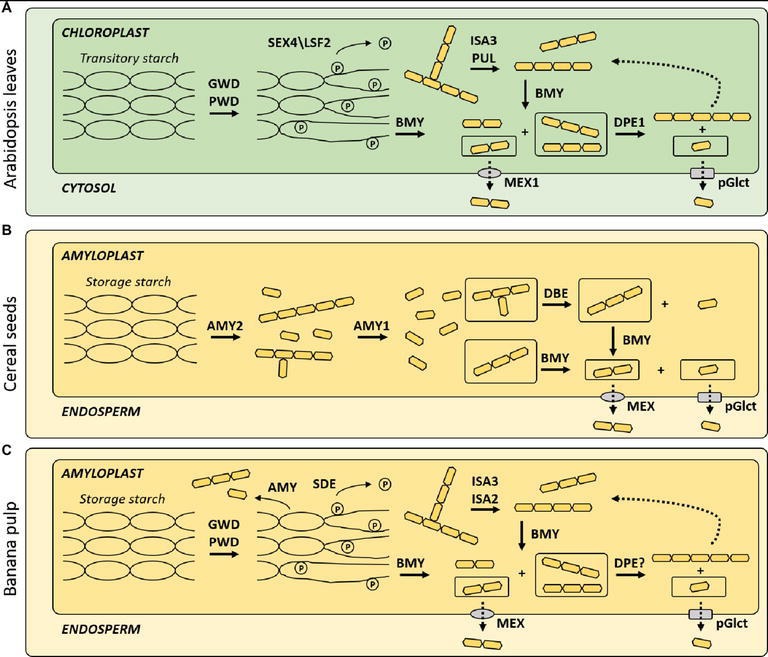
Starch-to-sucrose metabolism in model systems and banana pulp. Main enzymes responsible for starch degradation in **(A)** Arabidopsis leaves, **(B)** germinating cereal seeds, and **(C)** banana pulp. GWD, glucan, water dikinase; PWD, phosphoglucan, water dikinase; ISA, isoamylase-like protein sub-family; PUL, limit dextrinase family; BMY, β-amylase; DPE1, starch-disproportionating enzyme 1; MEX1, maltose excess transporter 1; pGlcT, plastidic glucose translocator; AMY, α-amylase.

Taxonomically, the banana is a commelinoid monocot (Musaceae) and, therefore, is more closely related to cereal grasses than *Arabidopsis* (Brassicaceae). However, although highly heterogeneous, the starch breakdown during banana ripening appears to be more akin to the process in *Arabidopsis* leaves (Figure 2A) than that of endosperm from germinating cereal seeds (Figure 2B). In photosynthetic tissues, the assimilation of energy through the Calvin cycle results both in carbon transference for sucrose synthesis in the cytosol and the production of transitory starch within the chloroplasts, which is a short-term energy supply when the tissue is not able to perform photosynthesis (Santelia and Lunn, 2017). In the dark, this transitory starch is converted mainly into glucose and maltose in the chloroplast, which are shipped to the cytosol for the synthesis of sucrose that can be further transported through the phloem to sink tissues (Pfister and Zeeman, 2016).

The starch stored in banana pulp cells is compartmentalized within plastids, similar to the transitory starch stored in the chloroplasts of *Arabidopsis* leaves. Studies have shown the presence and activity of several common starch-degrading enzymes (Figure 2C). In bananas, both AMY (e.g., MAmy) and β-amylases (BMY, EC 3.2.1.2) (e.g., bAmy) were associated with the starch granules in the amyloplasts of banana pulp (Peroni-Okita et al., 2013). A plastidic AMY identified in the banana (Junior et al., 2006) appears to act before BMY at the onset of starch breakdown, but the latter is essential for the complete breakdown and its upregulation is clearly correlated to a decrease in starch during fruit ripening (Purgatto et al., 2001). Alpha-amylase (AMY, EC 3.2.1.1) isoforms appear to play a crucial role in hydrolysis of starch in germinating cereal seeds (Radchuk et al., 2009) and is also present in the chloroplast of *Arabidopsis* leaves. However, AMY from *Arabidopsis* leaves, which have a strong preference for β-limit dextrin over amylopectin (Seung et al., 2013), is not necessary for the breakdown of transitory starch (Yu et al., 2005).

In *Arabidopsis* leaves, degradation of transitory starch at the end of the light cycle induces a transition from highly ordered to less ordered and hydrated granules by a complex process that involves the action of several starch-degrading enzymes. First, a group of starch-phosphorylating enzymes, termed glucan, water dikinase (GWD, EC 2.7.9.4), phosphorylates the C6 position of the glucosyl residues in starch (Ritte et al., 2006), and the steric hindrance of these (few) phosphorylated groups alters the intermolecular organization of the granule. This loss of structure favors the action of another group of starch-phosphorylating enzymes, termed phosphoglucan, water dikinase (PWD, EC 2.7.9.5) (Edner et al., 2007; Fettke et al., 2009), which acts downstream to GWD and phosphorylates the C3 position of the glucosyl residues. The role of phosphorylases including GWD and PWD in starch breakdown during banana ripening is less understood, but phosphorylation at the C3 and C6 position of the glucosyl residues in the starch of freshly harvested unripe bananas has already been found, as well as the presence of PWD and GWD associated with the granule through a proteomic analysis (Helle et al., 2018). Therefore, it is likely that GWD- and PWD-induced phosphorylation of banana starch favors granule hydration and phase transition from the crystalline state to the soluble state.

In *Arabidopsis* leaves, the neutral and phosphorylated glucans released from the granule surface undergo a complex net of enzymatic reactions. Starch-dephosphorylating enzymes prevent phosphate groups from obstructing the action of other enzymes, while starch-debranching enzymes from both the isoamylase-like protein 3 sub-family (DBE\ISA3, EC 3.2.1.68) and the limit dextrinase family (DBE\PUL, EC 3.2.1.142) hydrolyze side chains at the C6 position (Streb et al., 2012).

The breakdown of starch granules in amyloplasts during banana ripening is assumed to occur in a two-step process. First, AMY acts mainly in amylose-rich regions of the starch granule (amorphous lamella), thereby exposing amylopectin-rich regions (crystalline lamella). Then, the phosphorylation of residues at C3 and mainly C6 by PWD and GWD, respectively, favors the action of BMY. However, even if the correlation between BMY expression and starch breakdown is well established (Nascimento et al., 2006), other hydrolytic enzymes, such as a DBE\ISA3 (Maisa) with preference for β-limit dextrin (Bierhals et al., 2004), two DBE\ISA2 (Jourda et al., 2016), as well as starch phosphorylases (Mota et al., 2002) appear to contribute to starch degradation during ripening.

In photosynthetic tissues, the residual maltotriose produced by BMY (Li et al., 2017) can be used as substrates for starch disproportionating enzyme 1 (DPE1; EC 2.4.1.25), which transforms two molecules of maltotriose into glucose and maltopentaose (Critchley et al., 2001), and the latter could be a further target for BMY hydrolysis within the chloroplast (Moller and Svensson, 2016). Although the role of DPE is poorly understood in ripening bananas, genes encoding this enzyme were also found in the banana genome sequence^1^ suggesting they may be acting at the mobilization of starch during ripening. Finally, maltose and lesser amounts of glucose are shipped from the chloroplasts of the photosynthetic tissues to the cytosol mainly through the maltose excess transporter 1 (MEX1) and the plastidic glucose translocator (pGlcT), respectively (Cho et al., 2011). In the cytosol, sucrose phosphate synthase (SPS, EC 2.4.1.14) (Bahaji et al., 2015) and mainly sucrose phosphate phosphatase (SPP, EC 3.1.3.24) appear to form complexes that are crucial for sucrose synthesis (Albi et al., 2016). In bananas, it is very likely that the resulting glucose and maltose are shipped from the amyloplast to the cytoplasm through MEX and pGlcT in a mechanism similar to that of *Arabidopsis* leaves. Then, sucrose is synthesized mainly by SPS, which increases in activity during ripening, mainly by transcriptional activation (Nascimento et al., 1997a,b; Rossetto et al., 2003).

## Hormonal and Genetic Regulation of Starch-to-Sucrose Metabolism in Bananas

Ethylene is the most strongly hormone correlated to fruit ripening, particularly in climacteric fruits such as bananas. Climacteric ethylene synthesis in bananas induces the conversion of starch to soluble sugars (Saraiva et al., 2018), particularly sucrose, whereas the treatment with ethylene inhibitor, 1-methylcyclopropene (1-MCP), delays such conversion over several days in a dose- and cultivar-dependent manner (Mainardi et al., 2006; Nascimento et al., 2006). In addition, there are several indications that other hormones are associated and form a network of signals that coordinate the phases during fruit development (Seymour et al., 2013).

In recent years, the increasing availability of transcript profiling tools and *in silico* genomic analysis has allowed for the identification of several transcripts of enzymes related to starch mobilization in bananas that were affected by ethylene during ripening. Jourda et al. (2016) have identified four *DBE*, 13 *AMY* and 13 *BAM* by *in silico* analysis of the *Musa* genome. They have found that the transcripts of three *DBE*, five *AMY*, and three *BAM* are expressed in several stages of fruit ripening after treatment with exogenous ethylene, while two *BAM* (*MaBAM6* and *MaBAM7*) were induced after 24 h of acetylene treatment. Miao et al. (2016) have identified 16 members of the *MaBAM* family after *in silico* analysis of *Musa* genome, and 10 *MaBAM* transcripts were observed at various stages of development and ripening in bananas of cv. BaXi Jiao and cv. Fen Jiao. The genes encoding isoforms *MaBAM9a*, *MaBAM9b*, and *MaBAM3c* showed high levels of relative expression after climacteric in both cultivars.

Recently, an extensive study (Xiao et al., 2018) has found 38 genes associated with starch metabolism in bananas including three GWD, three phosphoglucan phosphatases, eight BMY, seven AMY, two DBE, two α-glucan phosphorylases, two DPE, two MEX (*MaMEX1* and *MaMEX2*), and five pGlct. Among these, 17 presented high transcript accumulation in ethylene-treated fruits (*MaGWD1, MaSEX4, MaLSF2, MaBAM2-MaBAM4, MaBAM6-MaBAM8, MaAMY3, MaAMY3B, MaAMY3C, MaISA3, MaMEX1, MapGlcT2-1, MapGlcT4-1*, *and MapGlcT4-2*). The same pattern was also observed at the climacteric of naturally ripening bananas and in fruits treated with 1-MCP. However, the fruits treated with 1-MCP were long delayed in relation to those allowed to ripen naturally, as ripening took place only after the increase of endogenous ethylene synthesis.

Using a quantitative proteomic approach, in the same work, the authors have identified 18 proteins related to starch degradation in protein extracts isolated from starch granules of unripe and ripe bananas. Among them, MaGWD1, MaPWD1, MaSEX4, MaLSF1, MaBAM4, MaBAM7, MaAMY2B, MaAMY2C, MaAMY3, and MaISA3 had higher levels in the extracts isolated from ripe than those from unripe fruits. A similar trend was observed in their transcript levels, as confirmed for the MaGWD1 protein by Western blotting using an anti-GWD1 antibody. Moreover, MaGWD1 accumulation was accelerated in ethylene-treated bananas and delayed after 1-MCP treatment.

The ethylene dependence of the gene expression of enzymes related to starch degradation was also observed in cold stored cv. Nanicão bananas, as the activity of BMY was reduced in fruits of that cultivar that had low levels of ethylene (Peroni-Okita et al., 2013). Thus, there is little doubt that ethylene is a hormone directly related to the activation of the starch mobilization system during banana ripening. In addition, the hormone appears to stimulate the activity of enzymes related to the sucrose metabolism, such as SuSy, SPS, and acid and neutral INV (Nascimento et al., 1997a; Choudhury et al., 2008; Li et al., 2011).

Although the evidence points to a significant role for ethylene in the regulation of starch mobilization in bananas, there is a lack of systematic analysis of ethylene response elements in the promoter regions of genes associated with starch metabolism in the *Musa* genome. Miao et al. (2017) have identified response elements to diverse hormonal classes in the upstream regions of 16 *MaBAM* genes. Interestingly, only *MaBAM3c*, which was highly expressed in the fruit after the climacteric peak, showed a single element of ethylene response (ERE) in the promoter region. This gene also had elements of response to auxin, abscisic acid, and methyl jasmonate in the promoter region, suggesting a multi-hormonal regulation of expression.

**Figure 3:**
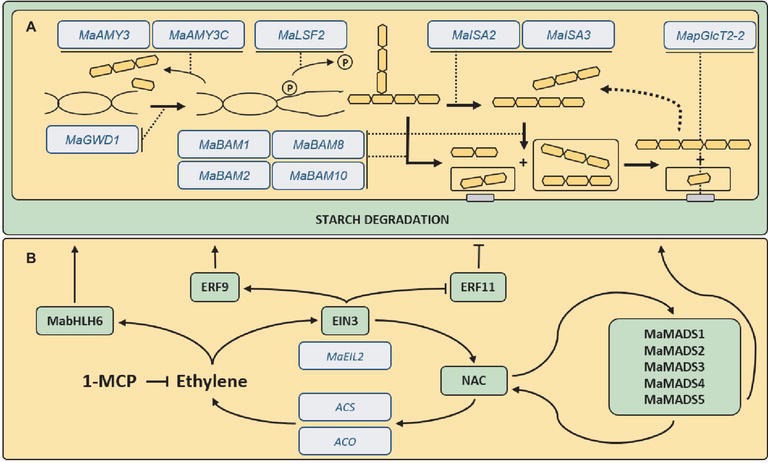
Ethylene-induced regulation of starch degradation. **(A)** A dual feedback loop of MADS-box sequences (MaMADS) and no apical meristem (NAM), *Arabidopsis* transcription activation factor 1/2 (ATAF), and cup-shaped cotyledon (CUC) (NAC) transcription factors appear to regulate ethylene responses in bananas. This results in the **(B)** induction of several genes related to starch degradation during fruit ripening including a glucan, water dikinase (Ma*GWD1*), six α- and β-amylases (*MaAMY3*, *MaAMY3c*, *MaBAM1*, *MaBAM2*, *MaBAM8*, and *MaBAM10*), two isoamylases (*MaISA2* and *MaISA3*), and a plastidic glucose translocator (*MapGlcT2*-*2*).

The analysis of promoter regions of other genes may also contribute to the understanding of the mechanism by which ethylene regulates starch mobilization in bananas. More than 200 AP2/EREBP transcription factors (TF) were identified in the *Musa* genome (D’Hont et al., 2012), indicating a high number of candidates for downstream effectors of ethylene signaling regarding the expression of starch-related enzymes, such as the TF named ethylene response factors (ERF). Ethylene-signaling activation starts with its recognition by a family of five ethylene receptors (ETR1, ETR2, ERS1, ERS2, and EIN4), which regulate downstream proteins such as CTR1, EIN2, EIN3, and EIN5. EIN3 (ethylene insensitive 3) is a nuclear-localized protein that acts as a TF by activating the ERF through binding to primary ERE (Liu et al., 2015). Mbeguie-A-Mbeguie et al. (2008) have isolated five *EIN3-like* genes from bananas, named *MaEIL* (*Musa acuminata ethylene insensitive 3-like*) from 1 to 5. All of them were differentially expressed during ripening with *MaEIL2* being the exclusive gene induced after ethylene treatment (Figure 3A). These TF regulate banana ERF, culminating in the expression of ripening-related genes such as the ones involved in starch-to-sucrose metabolism (Figure 3B).

A study of 15 banana *ERF* genes in ethylene- and 1-MCP-treated fruits found that *MaERF9* was upregulated, and *MaERF11* was downregulated in peel and pulp during ripening or after ethylene treatment (Yu et al., 2013). MaERF9 protein was considered to disrupt ethylene burst during banana ripening (with high starch degradation), while MaERF11 was a repressor of banana ripening (with low starch degradation) through direct and indirect mechanisms of action (Han et al., 2016). It can be deduced that the direct effect was caused by MaERF11 binding to the promoters of several ripening-related genes inhibiting their expression. The indirect effect was due to the recruitment of a banana histone deacetylase (MaHDA1) that alters the H3 and H4 histone acetylation levels during fruit ripening, reinforcing the repression of the ripening-related genes, until *MaERF11* expression decreases at the onset of ripening, triggering all the downstream events (Han et al., 2016).

Xiao et al. (2018) have found binding elements of bHLH (basic helix-loop-helix) TF in the upstream regions of 27 genes related to the starch mobilization in bananas. A transcription factor designated MabHLH6 demonstrated binding capacity to the promoter region of 11 genes and activated the transient expression of the luciferin reporter gene in tobacco leaves. These findings indicate that MabHLH6 is a direct regulator of several genes encoding enzymes from the starch mobilization pathway. In naturally ripened fruits, both transcript and MabHLH6 protein accumulated after the climacteric. Ethylene-treated bananas showed that MabHLH6 expression was hastened, and treatment with 1-MCP delayed the induction of MabHLH6 expression. This suggests that MabHLH6 regulation is ethylene-dependent, reinforcing the hypothesis that starch degradation in bananas could be mediated by TF from families other than the AP2/EREBP (Xiao et al., 2018).

Although they are still poorly explored, there are indications that auxins and gibberellins also exert an influence on starch-to-sugar metabolism in bananas. In fact, the role of plant hormones other than ethylene on the regulation of gene expression in climacteric and non-climacteric fruits shows that ripening is a net result of hormonal inputs in response to developmental factors as well as environmental signals (Seymour et al., 2013). It has been demonstrated, for instance, that tomato SlARF4, an auxin response factor, negatively regulates starch synthesis during tomato development (Sagar et al., 2013). Regarding banana fruit, previous studies (Purgatto et al., 2001) have indicated that auxin treatment delays starch mobilization with impact on sugar synthesis, and such observations were related to the downregulation of a BAM isoform. Interestingly, auxin treatment negatively affected beta-amylolytic activity without affecting sucrose synthetic activity, indicating that the delay on sugar accumulation is mostly due to impairment in starch mobilization (Purgatto et al., 2001).

Treatment with gibberellic acid also promoted a delay on starch degradation in bananas (Rossetto et al., 2003), although the mechanism is apparently not the same as for auxin. Studies with other hormones such as abscisic acid and methyl jasmonate have influenced banana ripening in other aspects, such as cell wall metabolism (Lohani et al., 2004) and carotenoid synthesis (Kaur et al., 2017). However, more research needs to be carried out to fully understand the cross talk of these plant hormones in starch-to-sucrose metabolism during banana ripening.

Another class of TF involved in starch metabolism in bananas is the MADS-box sequences (Mini-chromosome maintenance deficient 1–MCM1, AGAMOUS, DEFICIENS, and Serum response factor–SRF). The *MaMADS1* and *MaMADS2* genes, which were overexpressed during ripening of cv. Grand Nain (Cavendish, genotype group AAA), shared 49 and 54% homology to the *LeRIN-MADS* (*RIPENING INHIBITOR*), a MADS gene from the tomato, respectively (Friedman et al., 2007). Elitzur et al. (2010) have further observed that the expression of *MaMADS2*, *3*, *4*, and *5* genes increased before ethylene peak, while *MaMADS1* expression was observed together with the ethylene peak. *MaMADS3*, *4,* and *5* expressions were induced by ethylene treatment. Intriguingly, 1-MCP treatment at the onset of the ethylene burst also increased the expression of *MaMADS4 and MaMADS1,* suggesting two independent programs occurring throughout banana ripening with special roles for *MaMADS1 and MaMADS2* (Elitzur et al., 2010). According to Roy Choudhury et al. (2012), MaMADS5 protein interacts with the promoters of banana ripening genes, such as *MaSPS,* stimulating their expression. A bioinformatics study has shown that MaMADS24 and MaMADS49 proteins can interact with several other *MaMADS* genes and with genes directly related to banana ripening, such as the hormone-response and ethylene-signaling genes, as well as starch degradation-related genes (Liu et al., 2017). Recently, Lü et al. (2018) have demonstrated that the banana tree has undergone genome duplications that were responsible for a unique TF dual-positive feedback loop circuit for regulating fruit ripening and, consequently, starch degradation. The circuit was derived from a no apical meristem (NAM), *Arabidopsis* transcription activation factor 1/2 (ATAF), and cup-shaped cotyledon (CUC) (NAC) TF and the three *MADS* transcription factors described above (*MaMADS* 1, 2, and 5).

The effects of hormones and TF on the starch-to-sugar metabolism in bananas continue to be a field for further research. Further studies may add more layers of complexity to the understanding of the regulation of this pivotal metabolism in the physiology of the fruit, in addition to enabling the enhancement of its commercial quality.

## Data Availability

All datasets generated for this study are included in the manuscript.

## Author Contributions

All authors equally collected literature data, wrote the manuscript and revised the article.

### Conflict of Interest Statement

The authors declare that the research was conducted in the absence of any commercial or financial relationships that could be construed as a potential conflict of interest.

## Footnotes

^1^http://banana-genome.cirad.fr/
